# Fibrosis—the tale of H3K27 histone methyltransferases and demethylases

**DOI:** 10.3389/fcell.2023.1193344

**Published:** 2023-07-05

**Authors:** Morgan D. Basta, Svetlana Petruk, Alexander Mazo, Janice L. Walker

**Affiliations:** ^1^ Department of Pathology and Genomic Medicine, Thomas Jefferson University, Philadelphia, PA, United States; ^2^ Department of Biochemistry and Molecular Biology, Thomas Jefferson University, Philadelphia, PA, United States; ^3^ Department of Ophthalmology, Thomas Jefferson University, Philadelphia, PA, United States

**Keywords:** fibrosis, myofibroblast, extracellular matrix, chromatin, epigenetics, EZH2, EZH1, UTX

## Abstract

Fibrosis, or excessive scarring, is characterized by the emergence of alpha-smooth muscle actin (αSMA)-expressing myofibroblasts and the excessive accumulation of fibrotic extracellular matrix (ECM). Currently, there is a lack of effective treatment options for fibrosis, highlighting an unmet need to identify new therapeutic targets. The acquisition of a fibrotic phenotype is associated with changes in chromatin structure, a key determinant of gene transcription activation and repression. The major repressive histone mark, H3K27me3, has been linked to dynamic changes in gene expression in fibrosis through alterations in chromatin structure. H3K27-specific homologous histone methylase (HMT) enzymes, Enhancer of zeste 1 and 2 (EZH1, EZH2), which are the alternative subunits of the Polycomb Repressive Complex 2 (PRC2) and demethylase (KDM) enzymes, Ubiquitously transcribed tetratricopeptide repeat, X chromosome (UTX), and Lysine demethylase 6B (KDM6B), are responsible for regulating methylation status of H3K27me3. In this review, we explore how these key enzymes regulate chromatin structure to alter gene expression in fibrosis, highlighting them as attractive targets for the treatment of fibrosis.

## 1 Introduction

Fibrosis is a devastating disease for which there are currently no effective therapies. Fibrosis, or excessive scarring, is characterized by an uncontrolled accumulation of fibrotic extracellular matrix (ECM) that can occur in nearly any organ of the body (Herrera et al., 2018; Distler et al., 2019; Henderson et al., 2020). This accumulation of fibrotic ECM can have detrimental effects on organ function. Alpha-smooth muscle actin (αSMA)-expressing myofibroblasts are the cell type largely responsible for producing excessive ECM (Hinz, 2016). These αSMA-expressing myofibroblasts differentiate from various types of precursor cells depending on the tissue of origin (Hinz, 2016). The fibroblast is the most recognized myofibroblast precursor cell across multiple tissue types (Hinz, 2007; Micallef et al., 2012). Other myofibroblast precursor cells that have been discovered across distinct tissue types include fibrocytes, epithelial cells that undergo an epithelial to mesenchymal transition (EMT), mesenchymal stem cells, and pericytes (Hinz et al., 2012; Micallef et al., 2012). However, some myofibroblast precursor cells are organ specific. For instance, in liver fibrosis, hepatic stellate cells can serve as myofibroblast precursor cells, and in kidney fibrosis, mesangial cells can transition to myofibroblasts (Micallef et al., 2012). Despite the differences in tissue of origin and precursor cell type, the major characteristics of fibrosis remain the same. Matrix producing cells, namely, αSMA-expressing myofibroblasts, drive an abundance of ECM production, predominantly Collagen I and Fibronectin Extra Domain A (FN-EDA) splice form (Hinz, 2016; Herrera et al., 2018). These fundamental features of fibrosis are responsible for changes in the tissue environment that have deleterious effects on the tissue or organ.

**FIGURE 1:**
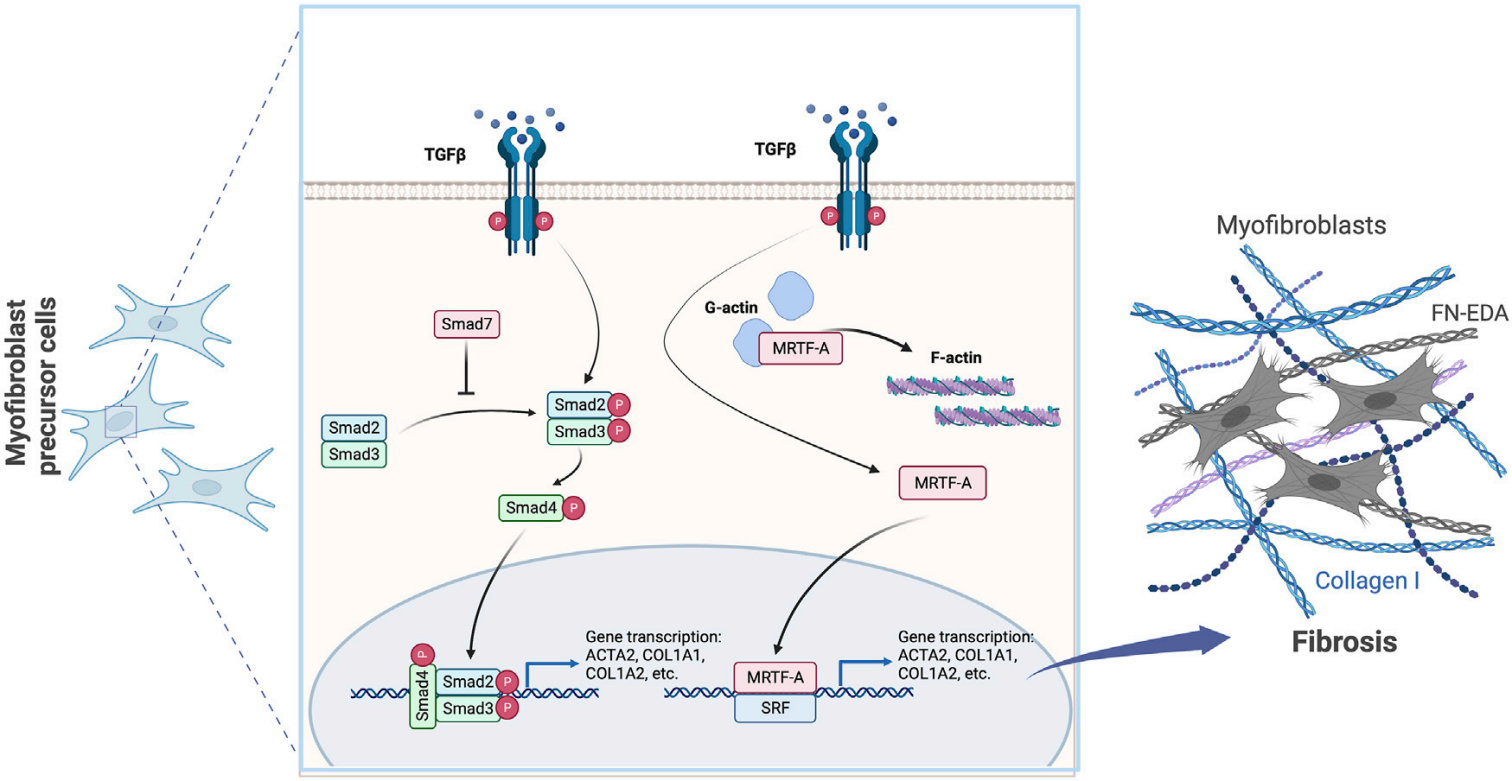
TGFβ is a central coordinator of signaling fibrosis. TGFβ signals through canonical Smad2/3 and/or noncanonical effectors leading to the activation of MRTF-A to drive emergence of myofibroblasts and the production of fibrotic ECM.

Classically, the fibrotic process in almost all cell and tissue types is governed by the activation of the transforming growth factor beta 1 (TGFβ1) signaling pathway (Hinz, 2016; Meng et al., 2016). TGFβ induces activation of regulatory Smads (R-Smads), Smad2/3 (mothers against decapentaplegic homologs 2 and 3) that associate with Smad4 (mothers against decapentaplegic homolog 4) to translocate to the nucleus to drive fibrotic gene expression (Biernacka et al., 2011). An important negative regulator of TGFβ signaling, that is typically downregulated during fibrosis, is the inhibitory Smad (I-Smad) Smad7 (mothers against decapentaplegic homolog 7) (Biernacka et al., 2011). Smad7 interacts with TGFβ type I receptor to block the association and phosphorylation of the receptor regulated Smad2/3 and Smad4 (Biernacka et al., 2011). TGFβ can signal through the canonical Smad pathway and through noncanonical pathways such as Rho GTPases (Biernacka et al., 2011). In addition to Smad proteins, other key pro-fibrotic transcription factors (TFs) downstream of TGFβ are myocardin-related transcription factor A (MRTF-A) and its cofactor, serum response factor (SRF) (Johnson et al., 2014; Korol et al., 2016; Werner et al., 2019). TGFβ-Rho- ROCK signaling is critical for the release of MRTF-A from globular actin (G-actin) by inducing filamentous actin (F-actin) polymerization to increase tissue stiffness, leading to the translocation of MRTF-A to the nucleus, where it associates with SRF to drive fibrotic gene expression (Hinz, 2009; Hinz, 2016; Tschumperlin et al., 2018). Together, MRTF-A and SRF are two major TFs downstream of TGFβ involved in the transcriptional regulation of major fibrotic genes (Gu et al., 2020; Sun et al., 2022) (Figure 1).

**FIGURE 2:**
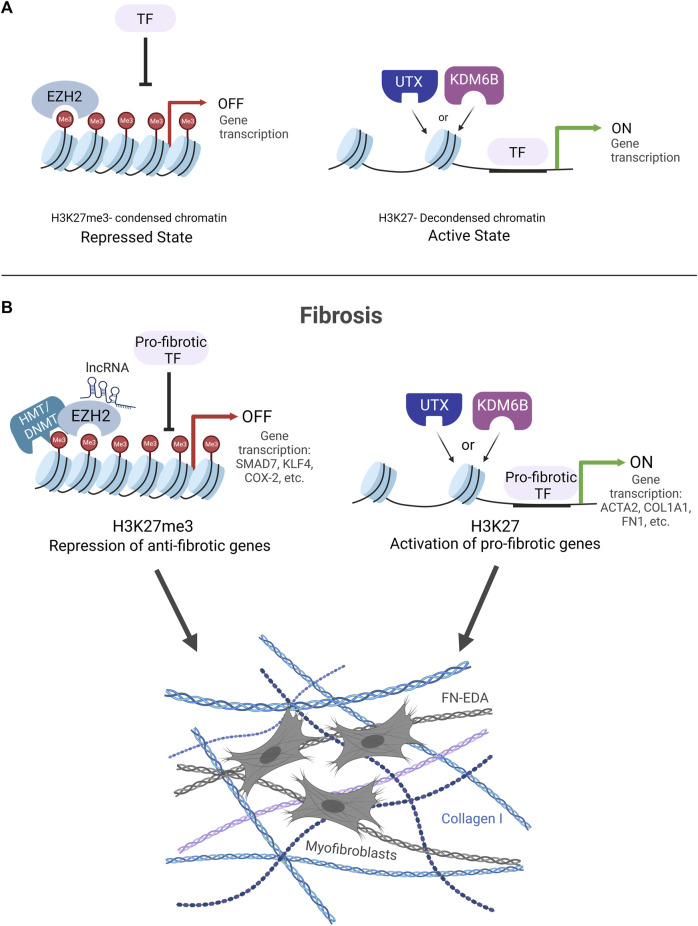
**(A)** H3K27me3 KDM and HMT enzyme regulation of chromatin structure. EZH2 methylates H3K27 to condense chromatin structure and block transcription factor (TF) binding leading to gene repression (Repressed State). UTX and KDM6B demethylate H3K27me3 to decondense chromatin structure to allow for TF binding and gene activation (Active State). **(B)**. Fibrosis is controlled by the dynamic interplay of EZH2 HMT and UTX/KDM6B KDM enzymes. EZH2 condenses chromatin structure to block TF binding on anti-fibrotic genes. Two potential mechanisms for how EZH2 silences anti-fibrotic genes are portrayed HMT/DNMT cooperation and lncRNA recruitment of EZH2 to gene promoters. UTX/KDM6B decondense chromatin structure to allow for TF binding and drive pro-fibrotic gene expression.

Growing research that spans the last decade demonstrates that fibrosis is regulated in large part by the dynamic methylation and demethylation of histone 3 lysine 27 (H3K27). Changes in the methylation of H3K27 influence alterations in chromatin structure, which are key to dictating gene expression through changes in the accessibility of regulatory factors to DNA. The methylation or demethylation of lysine 27 on the histone tail of H3 can lead to a condensed or decondensed structure of chromatin, respectively. The tri-methylation of H3K27 (H3K27me3) leads to a compact structure of chromatin characterized by a highly condensed array of nucleosomes (Bell et al., 2010; Yuan et al., 2012; Shlyueva et al., 2014). This compact organization prohibits the binding of TFs resulting in gene repression (Iwafuchi-Doi and Zaret, 2016) (Figure 2A). On the contrary, the demethylation of H3K27me3 decondenses the structure of chromatin through the loosening of nucleosomes, making it amenable to the binding of transcription factors to activate genes (Figure 2A).

H3K27 methylation and demethylation are regulated by two opposing types of histone modifying enzymes, HMTs and KDMs. Mono-, di-, and tri-methylation on lysine 27 of histone H3 (H3K27me1, H3K27me2, and H3K27me3) is catalyzed by the polycomb repressive complex 2 (PRC2) (van Mierlo et al., 2019; Chammas et al., 2020). The major enzymatic components of the PRC2 complex are EZH2 or EZH1, SET domain containing H3K27-specific HMT enzymes responsible for adding up to three methyl groups to the lysine 27 of histone 3 using the cofactor S-adenosyl-L-methionine (SAM) (Tan et al., 2014a; Piunti and Shilatifard, 2016). EZH1 or EZH2 are mutually exclusive within PRC2 complexes, and EZH1 compared to EZH2 is considered to have very little HMT activity (Margueron et al., 2008). Other components of the PRC2 complex required for regulating HMT activity are suppressor of zeste (SUZ12) and embryonic ectoderm development (EED) (van Mierlo et al., 2019; Chammas et al., 2020).

Opposing the PRC2-containing HMT enzymes are two KDM enzymes with H3K27me3-specific demethylase activity, UTX and KDM6B. These KDM enzymes are alpha-ketoglutarate-dependent hydroxylases that remove the methyl groups from H3K27me3 (Hong et al., 2007; Van der Meulen et al., 2014). It should be noted that a paralog of UTX with reduced KDM activity exists, Ubiquitously transcribed tetratricopeptide repeat, Y-linked (UTY) (Walport et al., 2014). These opposing HMT and KDM enzymes work in coordination to regulate the methylation and demethylation of H3K27, which is responsible for creating condensed and decondensed arrays of nucleosomes, respectively, and is essential to mediate gene expression (Figure 2A).

In this review, we describe how H3K27 methylase and demethylase enzymes influence H3K27-marked chromatin structure to alter gene expression to regulate fibrosis and whether they may represent essential therapeutic targets in fighting this disease.

## 2 Link between H3K27 HMT enzymes and fibrosis

### 2.1 Increased expression of EZH2 and high levels of H3K27me3 are associated with fibrosis

Among the H3K27me3-specific enzymes, EZH2’s role in fibrosis is the most extensively reported. The expression of EZH2 and its corresponding H3K27me3 mark are both remarkably increased in both human and experimental animal models of fibrosis relative to their non-fibrotic tissue/model counterparts. An increased expression of EZH2 was discovered in the following patient tissues: lung tissue from patients with idiopathic pulmonary fibrosis (Xiao et al., 2016), atrial muscle from patients with atrial fibrillation and fibrosis (Song et al., 2019), kidney tissue from patients with kidney disease associated with fibrotic scarring (Zhou et al., 2016), and dermal fibroblasts from patients with diffuse cutaneous scleroderma (Tsou et al., 2019). These important observations in real-world human disease are accurately portrayed in experimental animal models that mimic lung fibrosis (Xiao et al., 2016), liver fibrosis (Atta et al., 2014; Martin-Mateos et al., 2019), and kidney fibrosis (Zhou et al., 2016) adding value to experimental fibrosis models as promising predictors of human outcome. As expected, many of these same human and animal tissues/models that report increased EZH2 expression also report a concurrent increase in its major substrate, H3K27me3 (Xiao et al., 2016; Song et al., 2019; Tsou et al., 2019). The increased expression of EZH2 and the high amount of H3K27me3 in human fibrotic tissues and experimental animal models of fibrosis indicate a clear role for EZH2-induced gene repression in fibrosis.

### 2.2 The role of H3K27me3 HMT enzyme EZH2 in silencing anti-fibrotic genes to promote fibrosis

EZH2 function is linked to regulating fibrosis across many tissue types, including the liver (Mann et al., 2010; Yang et al., 2017; Argemi and Bataller, 2019; Martin-Mateos et al., 2019; Du et al., 2021; Jiang et al., 2021), lung (Xiao et al., 2016; Bao et al., 2021), kidney (Zhou et al., 2016; Zhou et al., 2018; Li et al., 2021c), heart (Zhu et al., 2016; Song et al., 2019; Ge et al., 2022), and skin (Tsou et al., 2019; Wasson et al., 2020). A major way that EZH2 may drive fibrosis is through EZH2-mediated methylation of H3K27 to repress anti-fibrotic gene expression. Smad7 and Dickkopf-related protein 1 (Dkk1) are classic anti-fibrotic genes associated with having a protective effect against fibrosis due to their ability to inhibit pro-fibrotic signaling through TGFβ (Biernacka et al., 2011; Meng et al., 2016) and Wnt (Akhmetshina et al., 2012; Wang et al., 2018), respectively.

TGFβ signaling is a key driver of the fibrotic process, and there are many points of regulation in this pathway (Figure 1) (Hinz, 2009; Biernacka et al., 2011). The major I-Smad, Smad7, blocks phosphorylation of R-Smads, activated downstream of TGFβ signaling, and subsequently prevents their translocation to the nucleus to inhibit activation of fibrotic genes (Biernacka et al., 2011). In a rat liver fibrosis model, bleomycin-induced lung fibrosis mouse model, and a rat unilateral ureteral obstruction (UUO) model for renal fibrosis, adenoviral or doxycycline-induced overexpression of Smad 7 blocked TGFβ signaling and suppressed fibrosis (Nakao et al., 1999; Dooley et al., 2003; Lan et al., 2003). This proof of concept was also shown in renal tubular epithelial cells (Li et al., 2002), within a dialysis stimulated peritoneal fibrosis rat model (Nie et al., 2007) and in a rabbit corneal injury model of corneal scarring (Gupta et al., 2017). On the contrary, deletion of the Smad 7 gene led to a worsened fibrotic response in a renal fibrosis UUO rat model (Chung et al., 2009) and within a mouse model to study inflammatory bowel disease (Schuler et al., 2023).

It was discovered that EZH2 has a role in repressing Smad7 to enable activation of fibrotic genes in cardiac, kidney, and peritoneal fibrosis. In cardiac fibroblasts, EZH2 can bind specifically to the Smad7 promoter to increase H3K27me3 and inhibit Smad7 expression (Ge et al., 2022). The therapeutic value of targeting EZH2 to relieve Smad7 repression and prevent TGFβ/R-Smad-induced fibrotic gene expression is substantial. Two inhibitors of EZH2 HMT activity, 3-Deazaneplanocin A (DZNep) and GSK126, can preserve Smad7 expression to significantly prevent UUO-induced kidney fibrosis, transverse aortic constriction surgery-induced cardiac fibrosis in mice, and a mouse model of peritoneal fibrosis (Zhou et al., 2016; Shi et al., 2020; Ge et al., 2022). Moreover, blocking EZH2 with DZNeP was able to upregulate Smad 7 expression, downregulate TGFβ signaling, and abrogate an EMT response associated with pulmonary fibrosis both in an *in vivo* LPS-induced acute respiratory distress syndrome-associated pulmonary fibrosis mouse model and in an *in vitro* model involving lung epithelial cells (Bao et al., 2021). All these data collectively support that EZH2 has a clear role in the TGFβ signaling pathway through its H3K27me3-induced repression of Smad7.

Another way that EZH2 can negatively regulate TGFβ signaling is through its ability to regulate the expression of BMP and Activin Membrane Bound Inhibitor (BAMBI), a TGFβ pseudoreceptor. BAMBI can prevent binding between type I and type II TGFβ receptors as well as form a ternary complex with Smad7 to cooperatively inhibit TGFβ signaling (Yan et al., 2009). In a study using both *in vitro* HSCs and *in vivo* carbon tetrachloride (CCL4) and bile duct ligation (BDL) liver mouse fibrosis models, treatment with DZNep to block EZH2 function led to upregulated transcription of BAMBI and blocked liver fibrosis (Jiang et al., 2021). Furthermore, in rat primary HSCs, DZNep treatment led to decreased recruitment of H3K27me3 upstream and downstream of the transcription start site of the *bambi* gene (Jiang et al., 2021). It will be of future importance to further unravel the molecular details underlying EZH2’s regulation of the TGFβ signaling pathway, both dependent and independent of Smad7 regulation.

Dkk-1 is a secreted negative inhibitor of the canonical Wnt/β-Catenin signaling pathway (Nishikawa et al., 2018), another major activator of fibrosis across many tissue and cell types (Guo et al., 2012; Tan et al., 2014b; Tao et al., 2016; Li et al., 2021b). For instance, Wnt can stimulate activation of liver hepatic stellate cells (Cheng et al., 2008; Nishikawa et al., 2018) and potently regulate systemic sclerosis (SSc) (Cheng et al., 2008; Akhmetshina et al., 2012; Daoussis et al., 2016; Henderson et al., 2021). Expression of the anti-fibrotic regulator Dkk-1 is reduced in both human and/or animal models of fibrosis, including SSc skin, pulmonary fibrosis, liver cirrhosis, and fibroblasts (Akhmetshina et al., 2012; Dees et al., 2014; Henderson et al., 2021). Upregulation of Dkk-1 expression in the skin of SSc patients due to treatment with rituximab to deplete B-cells led to an improved fibrotic condition (Daoussis et al., 2016). Furthermore, the addition of mesenchymal stromal cells could repair fibrosis in the lungs caused by radiation due to Dkk-1’s ability to block Wnt/β-catenin induced EMT (Shao et al., 2021) and adenoviral overexpression of Dkk-1 can block liver fibrosis in a BDL mouse liver model (Cheng et al., 2008). Dkk-1 can also block TGFβ induced Wnt activation and fibrosis in an experimental model of dermal fibrosis induced by adenoviral overexpression of a constitutively active TGF-β receptor type I (Ad-TBRI^act^) in the skin (Akhmetshina et al., 2012). All these studies provide strong evidence of Dkk-1’s potent anti-fibrotic activity through regulation of the Wnt/β-catenin signaling pathway.

Similar to Smad7 in cardiac fibroblasts, in a HSC cell line, EZH2 can tri-methylate H3K27 (H3K27me3) on the Dkk-1 promoter to repress Dkk-1 gene expression (Yang et al., 2017; Yang et al., 2022). The repression of Dkk-1 is considered an important component of HSC activation in the liver (Nishikawa et al., 2018). Conversely, inhibition of EZH2 HMT activity with DZNep or knockdown of EZH2 with small interfering RNA (siRNA) leads to inhibition of global H3K27 methylation levels and restoration of Dkk-1 expression (Yang et al., 2017). Restoration of Dkk-1 expression opposes Wnt/β-Catenin signaling to prevent HSC activation (Yang et al., 2017). It will be of future interest to understand whether Dkk-1 expression is under the control of EZH2 in other fibrotic tissue types associated with decreased expression of Dkk-1, such as systemic sclerosis of the skin (Henderson et al., 2021).

Another anti-fibrotic gene repressed by EZH2 is peroxisome proliferator-activated receptor gamma (PPARγ) (Deng et al., 2012), a ligand-activated transcription factor that is linked to promoting protective effects against lung, cardiac, kidney, and liver fibrosis (Kokeny et al., 2020; Wu et al., 2020; Han et al., 2021). In a UUO fibrotic mouse kidney model, PPARγ agonists have been effective in preventing TGFβ expression and interstitial fibrosis (Kawai et al., 2009). Activation of PPARγ is linked to the quiescent state of HSCs in the liver, preventing activation of HSCs into myofibroblasts and collagen expression (Hazra et al., 2004; Zhang et al., 2013). PPARγ agonists are effective against fibrosis across experimental models of liver fibrosis (Han et al., 2021) and are currently being used in clinical trials (Francque et al., 2021). In the liver, it was shown that PPARγ can oppose TGFβ-induced fibrosis. Crosstalk exists between these pathways, but the direct molecular mechanisms that link the two are far less understood. Skin fibroblast studies suggest that PPARγ can directly antagonize the Smad 3-mediated transcription response, which might help explain how PPARγ can oppose TGFβ signaling (Ghosh et al., 2004). Future studies are needed to fully understand the downstream relationship between PPARγ and TGFβ. However, the upstream silencing of PPARγ by EZH2 is far better understood. EZH2 has direct HMT activity on the PPARγ gene promoter to increase H3K27me3 levels, leading to repression of PPARγ (Li et al., 2018). Inhibition of EZH2 with DZNep has the opposite effect by allowing for de-repression and activation of PPARγ to block HSC activation and fibrotic gene expression in the liver (Mann et al., 2010; Li et al., 2018). Within HSCs, EZH2 can also reduce PPARγ expression by downregulating the PPARγ transactivator Kruppel-like factor 14 (KLF14) (Du et al., 2021). By ChIP analysis, EZH2 was shown to be recruited to the KLF14 promoter coincident with the accumulation of H3K27me3 (Du et al., 2021). Moreover, ectopic expression of KLF14 or inhibition of EZH2 with EPZ-6438 treatment could restore PPARγ expression and alleviate fibrosis in a rat thioacetamide liver fibrosis model (Du et al., 2021). This study highlights the ability of EZH2 to mediate transcriptional repression of anti-fibrotic genes at multiple levels.

Anti-fibrotic genes are recognized as important targets to protect against fibrosis, and the findings above strongly support the targeting of EZH2 function as an attractive strategy to preserve the expression of anti-fibrotic genes to block fibrosis across various tissue types.

#### 2.2.1 Mechanisms by which EZH2 can repress anti-fibrotic gene expression

There are two overarching mechanisms through which EZH2 can maintain a repressive state of transcription for the silencing of anti-fibrotic genes, to promote fibrosis: (1) EZH2 can cooperate with other histone methyltransferases and with DNA methylation binding proteins for specific silencing of anti-fibrotic genes, and (2) upstream RNA molecules can facilitate EZH2’s repression of anti-fibrotic genes.

EZH2 may work in coordination with methy-CpG-binding proteins and other methylation proteins to repress anti-fibrotic genes. In the liver, the previously mentioned, PPARγ, is silenced by the cooperation of EZH2 and the methyl-CpG-binding protein-2 (MeCP2). It was discovered that MeCP2 binds to the 5′ end of the PPARγ gene to drive H3K9 methylation and recruitment of the transcriptional repressor HP1α (Mann et al., 2010). The ability for EZH2 to increase H3K27 methylation on the PPARγ 3′ exon is coordinated by MeCP2 (Mann et al., 2010). It is proposed that MeCP2 can act as a signal to increase expression of EZH2 to enhance H3K27 methylation levels on PPARγ (Mann et al., 2010). This claim was supported by findings that show EZH2 expression is reduced upon MeCP2 siRNA silencing in myofibroblasts and within *Mecp2* ^*-/y*^ myofibroblasts (Mann et al., 2010). In addition, experiments blocking EZH2 function can increase PPARγ expression (Mann et al., 2010). From all these findings, the following mechanism of transcriptional repression of PPARγ was proposed: that transcriptional initiation is prevented by MeCP2 mediated H3K9 and HP1α recruitment to the 5′ end of PPARγ, and transcriptional elongation is prevented by EZH2 mediated H3K27 methylation at 3′exons of PPARγ (Mann et al., 2010). However, future studies are needed to understand how MeCP2 regulates EZH2 expression and whether this is a mechanism that is unique to liver fibrosis or perhaps it might explain a potential mechanism that can lead to increases in EZH2 expression typically observed in various other human and animal tissue models of fibrosis.

EZH2 works in coordination with the histone methyltransferase enzyme, H3K9 HMT G9a, to repress anti-fibrotic genes in lung fibrosis. The first study to examine a role for EZH2 and G9a in suppression of an anti-fibrotic gene assessed the selective silencing of Cyclooxygenase-2 (COX-2) (Coward et al., 2014). COX-2 drives the production of Prostaglandin E_2_ (PGE_2_), which has anti-fibrotic action on fibroblast activation and collagen production in the lung (Keerthisingam et al., 2001; Hodges et al., 2004; Coward et al., 2014). It was discovered in fibroblasts derived from the lungs of idiopathic fibrosis (IPF) patients that EZH2 leads to the tri-methylation of H3K27 and G9a tri-methylates H3K9, leading to condensed chromatin structure on the COX-2 promoter (Coward et al., 2014). It was also found that in addition to EZH2 and G9a-induced repression, DNMTs were also involved, leading together to histone hypermethylation and DNA methylation on the COX-2 promoter (Coward et al., 2014). This finding was later supported by the discovery that EZH2 and G9a also cooperate to regulate the repression of C-X-C motif chemokine ligand 10 (CXCL10) (Coward et al., 2018) another important factor in regulating pulmonary fibrosis (Tager et al., 2004). Together, these studies highlight that EZH2 and G9a can be dually targeted to relieve the H3K27me3 and H3K9me3-driven repression of anti-fibrotic genes in lung fibrosis. One area of caution is the specificity of these findings for lung fibrosis. CXCL10 has been shown to have pro-fibrotic roles in other tissue types of fibrosis, which suggests this mechanism might not be at play in other tissues (Hintermann et al., 2010; Guo et al., 2018; Chen et al., 2021). It will be interesting to determine whether MeCP2 and G9a also function in promoting fibrosis in distinct tissue types (Hu et al., 2011; Irifuku et al., 2016; Henderson et al., 2019; Ligresti et al., 2019) is linked to EZH2 as a common mechanism. These collective findings suggest that EZH2 might work in coordination with other histone and DNA methylation-dependent repressive proteins to epigenetically silence anti-fibrotic genes in certain contexts of fibrosis.

RNA molecules have emerged as upstream regulators of EZH2-induced repression of anti-fibrotic genes. Long noncoding RNA (lncRNA) and microRNA (miRNA) are two types of non-coding RNA molecules that have been identified as having a role as upstream regulators of EZH2. LncRNA molecules have the ability to recruit EZH2 to methylate H3K27 at the anti-fibrotic gene promoters (Yang et al., 2017; Ge et al., 2022; Yang et al., 2022). In cardiac fibrosis, EZH2 is recruited by the long noncoding RNA Nuclear enriched abundant transcript 1 (Neat1) to methylate H3K27 at the Smad7 promoter to inhibit its expression (Ge et al., 2022). Similarly, it was found that urothelial cancer associated 1 (UCA1) long noncoding RNA recruits EZH2 to the Dkk-1 promoter to repress Dkk-1 expression within hepatic stellate cells (Yang et al., 2022). These studies identify a role for lncRNA molecules to bind EZH2, recruit EZH2 to anti-fibrotic gene promoters, and increase H3K27me3 levels to promote fibrosis. miRNA molecules have a different role in the upstream regulation of EZH2. Overexpression of specific miRNA molecules in fibrosis can lead to a downregulation of EZH2 to relieve its repressive activity on anti-fibrotic genes. The repression of PPARγ by EZH2 can be alleviated in both cardiac and liver fibrosis through the overexpression of miR-214 and miR-29a, respectively (Zhu et al., 2016; Huang et al., 2019). In addition to the regulation of EZH2 expression by miRNAs, EZH2 can also be acetylated/deacetylated to impact its stability (Li et al., 2018). A histone deacetylase enzyme, Sirtuin 1 (SIRT1), can deacetylate EZH2 to decrease its stability and lead to the de-repression of PPARγ in liver fibrosis (Li et al., 2018). Taken together, the upstream regulation of EZH2 may be an essential part of the mechanisms by which EZH2 represses anti-fibrotic genes to promote fibrosis.

### 2.3 Non-canonical mechanisms by which EZH2 may promote fibrosis

A few studies indicate that EZH2 can act as a transcriptional activator of fibrotic gene expression, which may involve EZH2 interactions with the TGFβ signaling pathway. While EZH2 is largely described as a transcriptional repressor through its tri-methylation of H3K27 on gene promoters, there is also evidence that EZH2 can exhibit nonconventional functions outside of H3K27 methylation, including the ability to serve as a transcriptional co-activator (Kim et al., 2018; Wang and Wang, 2020; Huang et al., 2021). Since the major focus of this review is on how H3K27 modifying enzymes influence H3K27-marked chromatin structure to alter gene expression to regulate fibrosis, we will only briefly discuss these studies. In studies using an *in vitro* model for IPF that mimics epithelial remodeling, a noncanonical role for EZH2 was discovered as part of a transcriptional complex to direct an altered epithelial repair program (Le et al., 2021). In this study, nuclear transforming growth factor β activated kinase 1 (TAK1) was shown to cause phosphorylation of EZH2 to cause its release from the PRC2 complex to participate in a transcriptional complex with RNA polymerase II (RNA pol II) and nuclear actin to drive fibrotic gene expression (Le et al., 2021). A role for the catalytic function of EZH2 was established because treatment with GSK126 blocked the fibrotic response (Le et al., 2021). Furthermore, EZH2’s function in promoting atrial fibrosis was proposed to involve EZH2 forming a transcriptional complex with Smad protein on the ACTA2 promoter to activate ACTA2 gene expression (Song et al., 2019). Lastly, in a TGFβ stimulated iPSC-derived kidney organoid fibrosis model, an interaction was shown between EZH2 and Smad3 during myofibroblast differentiation (Davis et al., 2022). Interestingly, this study revealed that blocking EZH2 catalytic function with the GSK343 inhibitor led to a block in Smad3 dependent *cis* co-accessibility as well as a block in myofibroblast differentiation (Davis et al., 2022). However, how EZH2 and Smad3 specifically interact on chromatin to regulate gene expression to promote fibrosis is not clear. Overall, it is evident that EZH2 can play both a canonical role in repressing anti-fibrotic gene expression as well as noncanonical roles to promote a fibrotic response.

### 2.4 EZH2 has a context-dependent protective role in liver fibrosis

In contrast to EZH2’s role in promoting HSC activation for liver fibrosis, several studies suggest that EZH1 and/or EZH2 have protective roles in liver fibrosis. In three independent studies using mouse models of liver fibrosis, loss of EZH1 and EZH2 function led to the activation of fibrosis-related genes and the development or worsening of liver fibrosis (Bae et al., 2015; Grindheim et al., 2019; Lau-Corona et al., 2020). It is important to note that EZH2 was conditionally knocked out (cKO) using an abl-Cre promoter that specifically deletes EZH2 from the major parenchymal cells of the liver, the hepatocytes, but not from nonparenchymal cells, which include the HSCs and liver sinusoidal endothelial cells (LSECs). Furthermore, the EZH2 cKO was performed on an EZH1 global knockout background since it was necessary to delete both EZH1 and EZH2 because of compensation by one another (Bae et al., 2015; Grindheim et al., 2019). The mechanisms by which EZH1/EZH2 protect against liver fibrosis were shown to involve the action of these enzymes at euchromatic chromatin bivalently marked with H3K27me3/H3K4me3 at the pro-fibrotic gene promoters (Grindheim et al., 2019). Future studies are needed to determine the role of EZH1/EZH2 on euchromatic pro-fibrotic gene promoters, which may be independent of EZH2’s canonical role of condensing H3K27me3-marked chromatin structure to repress genes (Grindheim et al., 2019). In addition, EZH2 was described as playing a potential protective role in LSECs, a cell type important in maintaining HSC quiescence to help safeguard against fibrosis. EZH2 was found to interact with the long noncoding RNA, Antisense of IGF2R Non-Protein Coding RNA (Airn), which was postulated to prevent EZH2 from binding to KLF2, a transcription factor critical to promoting LSEC differentiation. In this scenario, EZH2 plays a role in maintaining the LSEC function to protect against HSC activation (Chen et al., 2022). Collectively, these studies highlight cell-context dependent roles for EZH1/EZH2 in the liver for regulating fibrosis and the need for liver cell-specific therapeutic strategies to target EZH1/EZH2 function to prevent fibrosis. Therapeutic promise to target EZH2 function in a cell context-dependent manner in the liver is provided by a study using an antibody-liposome-DZNeP that was targeted specifically to HSCs and found effective in blocking the fibrotic response in an *in vivo* CCl4-induced liver fibrosis mouse model (Zeybel et al., 2017).

## 3 Roles for UTX and KDM6B KDM enzymes to demethylate H3K27me3 to decondense chromatin and drive differentiation and fibrosis

While limited in the number of studies compared to EZH2 investigations, the H3K27me3 specific demethylase enzymes UTX and KDM6B have also been shown to promote fibrosis. UTX and KDM6B share similar roles in fibrosis, with only minor differences in their regulation of distinct cell and tissue types. In contrast to EZH2, which is considered to have predominant HMT activity relative to EZH1, there is strong evidence that both UTX and KDM6B share similar demethylase activity for H3K27me3 (Hong et al., 2007).

### 3.1 UTX and KDM6B as key drivers of cell differentiation

KDM6B and UTX have been shown to promote differentiation of various stem cells through the removal of histone methylation marks on H3K27me3, which can lead to activation of genes required for the acquisition of a new cell type (Xu et al., 2013; Petruk et al., 2017a; Petruk et al., 2017b). Using knockdown studies, KDM6B was shown to promote odontogenic differentiation of mesenchymal stem cells (MSCs) (Xu et al., 2013) and a poised pro-differentiation state in hematopoietic stem cells (Mallaney et al., 2019). KDM6B might also have a role in the activation and differentiation of immune cells such as B-cell subsets (Anderton et al., 2011) and CD8^+^ T cells (Li et al., 2021a). Similarly, UTX is essential for embryonic and hematopoietic stem cell lineage-specific differentiation (Petruk et al., 2017a; Petruk et al., 2017b), natural killer T cell development (Northrup et al., 2017), and germinal center B cell differentiation (Haniuda et al., 2020). It was also discovered that UTX has a role in lineage-specific retinal differentiation (Umutoni et al., 2020). Together, there is a well-documented ability for UTX and KDM6B to drive cell differentiation across many tissue types. It is reasonable to assume that UTX and KDM6B might have roles in driving precursor cell differentiation to myofibroblasts in fibrosis as well.

### 3.2 UTX and KDM6B demethylate H3K27me3 to promote fibrosis

Exploring the role of KDM6B and UTX in fibrosis, there is convincing evidence to suggest that demethylation of H3K27me3 promotes myofibroblast differentiation and fibrosis. A potent and selective dual inhibitor of KDM6B and UTX, GSK-J4, has shown therapeutic potential in emerging research by blocking the emergence of αSMA expressing myofibroblasts and the production of fibrotic matrix in various models of fibrosis (Neele et al., 2017; Lai et al., 2019; Hung et al., 2022). Inhibition of UTX/KDM6B lysine demethylase activity with GSK-J4 blocks transition to an αSMA-expressing myofibroblast in kidney fibrosis, lung fibrosis, and lens fibrosis (Hung et al., 2022; Basta et al., 2023). This finding is in line with the previously established role of UTX and KDM6B in promoting cell differentiation. Furthermore, it was discovered that GSK-J4 can suppress major ECM proteins such as collagen I, collagen III, collagen IV, fibronectin I, and fibronectin EDA in a variety of fibrosis types (Neele et al., 2017; Lai et al., 2019; Hung et al., 2022; Basta et al., 2023). It is proposed that UTX and KDM6B demethylate H3K27me3 to decondense nascent chromatin structure to allow TF binding to drive activation of pro-fibrotic genes (Basta et al., 2023). Perhaps inhibiting UTX and KDM6B to allow for the tri-methylation of H3K27 (H3K27me3) to prevent TF binding and activation of fibrotic genes (Basta et al., 2023) explains why GSK-J4 has such a potent effect on blocking fibrosis. Altogether, these studies reveal the KDM activity of UTX and KDM6B as key drivers of myofibroblast emergence and the production of fibrotic extracellular matrix.

There are some nuances to note regarding whether UTX or KDM6B have a predominant activity over the other in some fibrosis models/tissue types. Knockdown studies in peritoneal foam cells (Neele et al., 2017) and kidney fibrosis mouse models (Yu et al., 2021) point to KDM6B as a major driver of pro-fibrotic gene expression, the acquisition of a myofibroblast phenotype, and the production of fibrotic ECM. Furthermore, in an experimental model of systemic scleroderma a predominant role for KDM6B (JMJD3) was revealed in SSc fibroblasts (Bergmann et al., 2018). There are two additional studies that indicate a primary role for KDM6B in fibrosis. However, they contrast the previously outlined role of KDM6B in that they find that KDM6B has a protective role in kidney and liver fibrosis. Similar to the role of EZH2 in fibrosis, it is speculated that KDM6B regulates Smad7 expression because knockdown of KDM6B leads to a reduction in Smad7 expression in kidney fibrosis (Yu et al., 2021). Similarly, overexpression of KDM6B leads to an increase in Smad7 expression in liver fibrosis (Jiang et al., 2021). These findings might reveal a more complicated role for KDM6B, specifically in regulating fibrosis. Despite these two studies, both UTX and KDM6B have a clear role in activating pro-fibrotic genes to promote fibrosis.

## 4 Metabolic regulation of H3K27me3 HMT and KDM enzymes in fibrosis

An emerging area of research involves the connection between metabolism, fibrosis, and the discussed enzymes: EZH2, UTX, and KDM6B. Metabolism, specifically the conversion of glucose to pyruvate by glycolysis and the conversion of glutamine to α-ketoglutarate via glutaminolysis, have become major well-known drivers of fibrosis (Henderson and O'Reilly, 2021). There is a strong connection between the classic driver of fibrosis, TGFβ1 and an increase in these metabolic processes in pulmonary fibrosis, liver fibrosis, skin fibrosis, and systemic sclerosis (Henderson and O'Reilly, 2021). The phenotypic changes that accompany fibrosis, including myofibroblast differentiation and the accumulation of matrix proteins such as collagen I, are metabolically demanding (Gibb et al., 2020). An important role for these metabolic processes in fibrosis is supported by studies showing roles for glycolysis and glutaminolysis in driving fibrotic disease (Henderson and O'Reilly, 2021; Gibb et al., 2020). Interestingly, EZH2, UTX, and KDM6B are metabolically regulated, which raises the question of whether there is a connection between the metabolic regulation of these enzymes and metabolic changes in fibrosis.

Both UTX and KDM6B require α-ketoglutarate as a cofactor to hydroxylate the methyl groups on H3K27me3 (Agger et al., 2008). Therefore, α-ketoglutarate availability has the potential to influence the KDM activity of UTX and KDM6B. Indeed, it has been shown that intracellular α-ketoglutarate levels can influence KDM activity (Carey et al., 2015). The increase in glutaminolysis discovered in fibrosis would lead to an increase in the amount of α-ketoglutarate produced, and it was found that α-ketoglutarate is increased in lung myofibroblasts (Ge et al., 2018). It is a likely assumption that an increase in α-ketoglutarate in fibrosis would lead to increased UTX/KDM6B KDM activity. Human IPF fibroblast studies revealed the importance of glutamine in part through glutaminolysis and conversion to α-ketoglutarate and increased UTX/KDM6B KDM activity on survival genes and pro-fibrotic genes, including *COL3A1* (Bai et al., 2019; Xiang et al., 2023).

EZH2 HMT activity is also regulated by metabolism since it is dependent on SAM as a cofactor to methylate H3K27. Methionine is a precursor of SAM, and it has been shown in colorectal cancer cells and C57Bl6 mice that methionine depletion reduces the amount of SAM produced and consequently decreases the methylation of H3K27 by EZH2 (Zhang et al., 2020). The connection between methionine metabolism and EZH2 HMT activity in the context of fibrosis remains unclear and requires further investigation. However, it is well known that a methionine-choline-deficient diet (MCDD) in mice leads to liver fibrosis (Koca et al., 2008; Mu et al., 2010; Luo et al., 2013). Whether this is due to or related to EZH2 HMT activity remains to be discovered.

Together, the metabolic regulation of these enzymes: EZH2, UTX, and KDM6B might increase their activity in fibrosis. However, the exact connection between metabolism, fibrosis, and the activity of these enzymes remains to be thoroughly defined and requires future studies.

## 5 Conclusion

Treatment strategies aimed at H3K27-specific enzymes EZH2, UTX, and KDM6B are likely to have therapeutic potential for the treatment of fibrosis. The studies reviewed above provide evidence that the fibrotic genome is regulated by the dynamic interplay of these enzymes and their influence on H3K27-marked chromatin structure. A survey of the literature makes it apparent that there are major roles in fibrosis for EZH2 in silencing anti-fibrotic genes and UTX and KDM6B in promoting pro-fibrotic genes (Figure 2B). However, complicating this narrative are divergent studies showing the inverse, where inhibiting EZH2, UTX, and KDM6B can worsen fibrosis and may involve other H3K27- independent mechanisms. Clearly, more work is needed to fully understand these nuances when considering these enzymes as potential therapeutic targets to treat fibrosis. Of special note, we raise caution regarding studies that use DZNep to inhibit the HMT activity of EZH2 because DNZep inhibits the synthesis of S-adenosylhomocysteine (SAH) to inhibit EZH1/2 proteins rather than directly inhibiting the EZH2 catalytic site. It has been reported that DZNep globally inhibits histone methylation in cancer cells and is not selective for EZH2 (Miranda et al., 2009). The promise for the use of small molecular inhibitors to target UTX/KDM6B and EZH2 enzymes as an anti-fibrotic therapy is provided by the FDA-approved EZH2 inhibitor, tazemetostat, an oral treatment currently being used with cancer patients (Eich et al., 2020; Straining and Eighmy, 2022; Zauderer et al., 2022). In conclusion, continued efforts to determine how these enzymes work together as switches to methylate or demethylate H3K27, alter chromatin structure, and regulate gene expression will facilitate the development and optimization of future fibrosis therapies.
